# Influence of colloid infusion on coagulation during off-pump coronary artery bypass grafting

**DOI:** 10.4103/0019-5049.63653

**Published:** 2010

**Authors:** K Muralidhar, Rajnish Garg, SK Mohanty, Sanjay Banakal

**Affiliations:** Senior Consultant and Professor Anaesthesiology and Director (Academic), Narayana Hrudayalaya Institute of Medical Sciences, Bangalore, India; 1 Consultant Anaesthesiology, Narayana Hrudayalaya Institute of Medical Sciences, Bangalore, India; 2 Senior Consultant Anaesthesiology, Narayana Hrudayalaya Institute of Medical Sciences, Bangalore, India

**Keywords:** Cardiac, coagulation, hydroxyethyl starches, surgery, volume replacement, von Willebrand factor

## Abstract

This study was conducted to determine the influence of colloid infusion on coagulation in patients undergoing off-pump coronary artery bypass grafting (OP-CABG). Thirty patients undergoing elective OP-CABG received medium molecular weight hydroxyethyl starch group I (MMW-HES 200/0.5), low molecular weight hydroxyethyl starch group II (LMW-HES 130/0.4) or gelatin group III (GEL) in a prospective randomized trial. Blood samples were assessed for hemoglobin (Hb), activated coagulation time (ACT), prothrombin time (PT), activated partial thromboplastin time (aPPT), platelet count, fibrinogen and von Willebrand factor (vWF) at specified intervals. Total volume of the colloid infused and postoperative chest-time drainage was also measured. There was a significant decrease in Hb, platelet count, fibrinogen levels in all these groups, which did not warrant blood transfusion. After the colloid infusion, vWF decreased significantly to 67% from baseline in group I as compared to 85 and 79% in group II and group III, respectively. vWF levels remained lower than the baseline value in the first 24 hours in group I, whereas this factor level increased above the baseline values in groups II and III, 6 hours postoperatively. Postoperative chest tube drainage in 24 hours was significantly higher in group I (856 ± 131 ml) as compared to group II (550 ± 124 ml) and group III (582 ± 159 ml). LMW-HES 130/0.4 was superior to MMW-HES 200/0.5 and gelatin in patients undergoing OP-CABG, in terms of better preservation of coagulation associated with enhanced volume effect.

## INTRODUCTION

Off-pump coronary artery bypass grafting (OP-CABG) has revolutionized the surgical approach for the treatment of coronary artery disease. OP-CABG avoids the use of extracorporeal circulation and hence all the detrimental effects of instituting cardiopulmonary bypass, especially the systemic inflammatory response syndrome, are eliminated in patients undergoing this technique of coronary revascularization. Essential to the performance of OP-CABG is the use of mechanical stabilizers, e.g., octopus II and/or starfish, which make the distal anastomotic site immobile during grafting. In addition to mechanical stabilization, manipulation of the heart to provide access to the distal anastomotic site is necessary for optional visualization. The use of mechanical stabilizers and manipulation of the heart for optimal position prior to grafting make the haemodynamic state unsteady and are associated with a fall in cardiac output and systemic hypotension. One of the strategies employed to counter hypotension and decrease in cardiac output is the maintenance of crystalloid/colloid infusion to maintain optimal filling pressure and preload. Although the colloid crystalloid controversy still exists, colloids are usually utilized to prevent or treat hypovolemia in cardiac surgery. Colloids have the advantages of longer half-life than crystalloids and hence prolonged volume effects, maintenance of colloid oncotic pressure, and increasing the microcirculation.[1‐3] Colloid solutions remain a widely used therapy for preoperative maintenance of intravascular oncotic pressure and augmentation of plasma volume.[4] Among the colloids, gelatins and hydroxyethyl starches (HES) are the most commonly used.[5] However, these colloids may adversely affect the coagulation and primary haemostasis. Though gelatins are popular, there is a higher incidence of allergic reactions in comparison with HES. HES are derived from amylopectin which is a polysaccharide component of waxy maize. Impairment of homeostasis is most pronounced with large and highly substituted HES molecules such as hetastrach (HES 450/0.7) or medium molecular weight starches (MMW-HES 200/0.5) Voluven^®^ (fresenius Kabi, Bad Homburg, Germany). LMW-HES 130/0.4 is an HES preparation which has been developed to provide more osmotically effective small molecules to maintain effectiveness and to enhance metabolism and renal elimination of the substance, thereby improves drug safety.

Waitzinger *et al*. demonstrated a volume effect of LMW-HES 130/0.4 of about 100% of infused volume lasting for approximately 4–6 hours in healthy volunteers.[6,7] In contrast to other HES preparations, LMW-HES 130/0.4 does not accumulate in plasma following repeated doses. Though LMW-HES 130/0.4 is approved for the treatment of hypovolemia, its effects on coagulation in patients undergoing OP-CABG are not documented. The present study was designed to assess the influence of LMW-HES 130/0.4 on haemostasis during off-pump coronary revascularization and to compare its influence on haemostasis gelatin based solution (Gelofusine).

## METHODS

This study was designed as a prospective randomized controlled trial in patients undergoing elective first time off-pump coronary artery bypass surgery. The study was approved by the ethics committee of the hospital and written consent was obtained from the patients. Exclusion criteria included acute myocardial infraction, infarction within previous 3 months, poor left ventricular (LV) function (LV ejection fraction < 35%), renal insufficiency (serum creatinine > 2.0 mg%), liver impairment (ALT/AST > 40 U/l), anaemia (preoperative hematocrit < 35%), preexisting coagulopathy, platelet count of <150 × 10^3^/mm^3^, patients treated with heparin or cyclo-oxygenase inhibitors (e.g., aspirin) within last 7 days and history of allergy to colloids.

All anti-anginal and anti-hypertensive drugs were continued till the day of surgery. Pre-anesthetic medication consisted of oral diazepam 1 hour prior to anaesthesia. Continuous electro cardio gram(ECG) monitoring of leads I, II, V_5_ with automated ST segment analysis was initiated. Right femoral artery was cannulated under local anaesthesia for monitoring direct blood pressure and blood gas sampling. Anaesthesia was induced with a combination of midazolam, fentanyl and 1–2 mg/kg propofol and maintained with isoflurane in oxygen, fentanyl and midazolam. Endotracheal intubation was achieved after the administration of pancuronium bromide. A triple lumen central venous catheter was inserted in the right internal jugular vein for monitoring the central venous pressure, vasoactive drug infusion and blood sampling. Nitroglycerine (NTG) and dopamine infusions were titrated to maintain a mean arterial pressure within 20% of basal values. A maintenance fluid administration in the form of Ringer' lactate at 2 ml/kg/hour was infused an all patients. The central venous pressure was maintained between 10 and 14 mm Hg using colloid infusion as needed. OP-CABG was done after median sternotomy under normothermic conditions with cardiopulmonary bypass (CPB) stand by. Heparin was used in a dose of 300 units/kg to maintain an activated coagulation time (ACT) of at least 300 seconds. Proximal anastomosis was done with aortic partial clamping, and distal grafting was performed using octopus-II tissue stabilizer. OP-CABG was converted to conventional CABG with CPB, if the patient could not tolerate the procedure off-pump. After anastomoses, heparin was neutralized with protamine in a ratio of 1 mg protamine for every 100 units of initial dose of heparin. No antifibrinolytic agents were used. Cell saver and autologous blood transfusion were not a part of the study protocol. Blood and blood products were not used until hematocrit was <27%. Patients were electively ventilated at the end of surgery and early extubation protocol was applied to all the patients.

Patients were randomly allocated to three groups as follows: group I (MMW-HES 200/0.5) received 6% medium molecular weight hydroxylethyl starch 'Haes-steril^®^' (Fresenius Kabi, Pune, India), group II (LMW-HES-130/0.4) received 6% low molecular weight hydroxyethyl starch 'Voluven^®^' (Fresenius Kabi, Bad Homburg, Germany) and group III (GEL) received 4% succinylated gelatin 'Gelofusine^®^ (B Braun, Melsungen AG, Germany). The colloid was infused in a dose of 7–8 ml/kg prior to the administration of heparin and later the infusion rate was dictated by the clinical need. Blood samples were taken at the following timings: *T*_1_, prior to anesthetic induction; *T*_2_, after infusion of 7–8 ml/kg of colloid but before administration of heparin; T_3_, 6 hours postoperatively and T_4_, 24 hours postoperatively. Each sample was assessed for haemoglobin (Hb), ACT (MINI, Actalyke, TX, USA), prothrombin time (PT), activated partial thromboplastin time (aPTT) (photo optical method, bioMerieux, France), platelet count (automated analyzer), fibrinogen (photo optical method, bioMerieux, France) and von Willebrand factor (vWF) [enzyme-linked immunosorbent assay (ELISA) – Vidas, bioMerieux, France]. Postoperative chest tube drainage and total volume of colloid infused in first 24 hours was monitored. Data were expressed as mean ± standard derivation (SD) and parameters were analyzed statistically. Comparison with *T*_1_ at each interval (*T*_2_, *T*_3_, *T*_4_) was done by Wilcoxon's signed rank test. Multiple group comparisons were done by one-way analysis of variance (ANOVA) followed by Mann-Whitney test (alternative to unpaired *t*-test). A '*P*' value of <0.05 was considered to be statistically significant.

## RESULTS

There were 30 patients in the study group, 10 patients in each of the groups. Blood samples were assessed for Hb, ACT, PT, aPPT, platelet count, fibrinogen and vWF at four specified intervals. In addition, total volume of colloid infused and postoperative chest-time drainage was also measured. Though the sample number in each group was small, it was felt that statistically significant values for 'between' group/'within' group comparison could be obtained and this could be used as a pilot study for a larger study.

The demographic and preoperative details are given in Table 1. The demographic and preoperative data were comparable in the three groups. All patients completed the study and there were no conversions to 'on-pump' and there was no mortality.

**Table 1:** Demographic and other data (Mean ± SD)

	Group – I (MMW-HES 200/0.5) n = 10	Group – II (LMW-HES 130/0.4) n = 10	Group – III (GEL) n = 10
Age (Yrs)	54.1±11.3	59.3±7.1	61.4±5.9
Weight (kg)	70.7±9.8	66.6±8.4	65.6±7.4
Sex (M / F)	8/2	9/1	9/1
Diabetes mellitus	4	5	4
Hypertension	5	5	6
Previous MI	6	5	5
COPD	0	0	1
No	1-3(2)	1-3(2)	1-3(2)
Total volume infused (ml)	2200±307.3	1920±229.9•	2700±197.2•*
Post-operative blood loss (ml)	856±131.1	550±124.9•	582±159.0•

Values are mean ± SD (standard deviation); MI= myocardial infraction; COPD= chronic obstructive pulmonary disease,

• = Values differ significantly among groups I and II (significance set at *P*<0.05),

* = Values differ significantly among groups II and III (significance set at *P*<0.05)

There was a significant decrease in the Hb concentration and platelet count [Table 2] in all the groups when *T*_1_ was compared to *T*_3_ and *T*_4._ ACT measurements showed similar trend in all the groups with no significant change from initial values during the study period [Table 2]. PT was not significantly different from basal values in all the groups and in between groups [Table 2]. Likewise, PTT was similar within the group and in between groups [Table 2].

**Table 2:** Haematological values at various stages in three groups (mean ± SD)

Variable	Group-I (MMW-HES 200 / 0.5)	Group-II (LMW-HES 130/0.4)	Group-III (GEL)
Haemoglobin (gram/100ml)			
T_1_	14.1±0.8	13.9±0.5	13.9±0.6
T_2_	13.6±0.9	13.6±0.4	13.6±0.5
T_3_	10.7±0.7*	11.2±0.6*	11.1±0.6*
T_4_	10.4±0.7*	10.8±0.6*	10.5±0.4*
Platelet count (×10^5^/mm^3^)			
T_1_	2.4±0.6	2.4±0.6	2.3±0.4
T_2_	2.2±0.6	2.1±0.5	2.1±0.4
T_3_	1.7±0.4*	2.1±0.4	1.9±0.3
T_4_	1.9±0.4*	2.1±0.5	2.1±0.4
ACT (seconds)			
T_1_	114.8±4.2	117.0±5.2	114.3±6.0
T_2_	116.8±4.6	118.0±4.8	117.8±5.2
T_3_	112.9±5.4	112.9±4.7	107.8±6.9
T_4_	112.6±3.2	111.2±4.6	104.5±6.4
PT (seconds)			
T_1_	12.4±0.4	12.6±0.4	12.7±0.6
T_2_	13.4±0.5	14.1±0.2	13.4±0.6
T_3_	15.1±0.6	14.7±0.4	14.4±0.4
T_4_	15.7±0.5	14.8±0.5	15.0±0.4
aPTT (seconds)			
T_1_	28.0±1.0	28.0±1.9	28.0±1.0
T_2_	28.6±0.8	28.0±1.2	28.0±1.3
T_3_	29.4±1.6	28.0±1.8	28.6±0.8
T_4_	29.6±2.0	28.0±0.6	29.0±1.5
Fibrinogen (mg/dl)			
T_1_	312.6±53.8	354.8±54.4	334.7±48.4
T_2_	223.3±58.9*	278.9±43.2*	274.0±44.7*
T_3_	169.5±50.6*	220.5±27.3*	1229.5±44.7*
T_4_	208.7±49.4*	302.8±43.6*•	301.5±43.7*•
vWF(%)			
T_1_	210±67.6	235±54.8	197±83.5
T_2_	138±65.5*	201±58.9*	156±63.5*
T_3_	189±81.4*	390±101.3*•	269±96.0*•
T_4_	205±35.2	397±83.3*•	270±57.7*•

ACT= Activated clotting time; PT= Prothrombin time; PTT= partial thromboplastin time; VWF= von Willebrand factor, Values are mean ± SD (standard deviation),

• = Between group (significance set at *P*<0.05),

* = Within group (*P*<0.05)

Fibrinogen levels significantly decreased from baseline values in all the groups from *T*_1_ to *T*_3_ and there was recovery of fibrinogen levels at *T*_4_ in all the groups but this was still lower than basal values in all the groups. In between groups, fibrinogen levels were significantly lower in group I (MMW-HES 200/0.5) as compared to groups II (LMW-HES 130/0.4) and III (GEL). The plasma levels of vWF decreased significantly in group I (MMW-HES 200/0.5) from *T*_1_ to *T*_2_ and then showed recovery but plasma levels were lower than basal values at *T*_3_ and *T*_4_. In groups II (LMW-HES 130/0.4) and III (GEL), plasma levels of vWF decreased significantly at *T*_2_ from *T*_1_ and after recovery its levels increased significantly at *T*_3_ and *T*_4_ from basal values [Table 2]. In between groups, there was a significantly lower vWF level in group I as compared to groups II (LMW-HES 130/0.4) and III (GEL) at *T*_3_ and *T*_4_. vWF levels are significantly lower in group III (GEL) as compared to group II (LMW-HES 130/0.4) at *T*_3_ and *T*_4_ [Table 2].

Postoperative blood loss through the chest drainage tubes was significantly higher in group I (MMW-HES 200/0.5) as compared to groups II (LMW-HES 130/0.4) and III (GEL). In groups II (LMW-HES 130/0.4) and III (GEL), there was no significant difference in blood loss [Table 1]. Total volume of colloid infused was significantly higher in group III (GEL) as compared to groups I (MMW-HES 200/0.5) and II (LMW-HES 130/0.4). In group I (MMW-HES 200/0.5), volume infused was significantly more than in group II (LMW-HES 130/0.4) [Table 1]. There were no differences in haemodynamics/oxygenation/renal parameters in the three groups. None of the patients received blood/blood products and there were no re-explorations for excessive drainage in all the groups.

## DISCUSSION

This study demonstrates that LMW-HES 130/0.4 is superior to both MMW-HES 200/0.5 and gelatin solutions because use of LMW-HES 130/0.4 caused the least impairment of coagulation. The choice of colloids for volume infusion in cardiac surgery has been debated for several years.[8] Two of the major concerns involved in the use of synthetic colloids (e.g., HES or gelatin) are (i) alteration of coagulation and (ii) possibility of anaphylaxis.[9,10] Altered haemostasis and increased bleeding have been reported with the use of HES solutions in cardiac surgical patients. However, in majority of these reports, first-generation HES namely hetastrach (MW = 450,000 Da with high degree of substitution of 0.7) was used. Hetastrach has been reported to induce a type-I von Willebrand like syndrome with decreased factor VIII coagulant activity and decreased vWF antigen and factor VIII related ristocetin cofactor. Decreased interaction with factor VIII and vWF while using more rapidly eliminated hydroxyethyl starch types (LMW-HES 130/0.4) might translate to smaller blood losses and a reduced consumption of blood products. Langeron *et al*.[11] described a significantly reduced need for allogeneic transfusions when comparing LMW-HES 130/0.4 to MMW-HES 200/0.5 (*P* = 0.042) infused at the same dose levels. Significantly less interference with factor VIII concentrations 5 hours after the end of surgery was seen in the LMW-HES 130/0.4 group (*P*<0.05). Gallandat Huet *et al*.[12] found significantly lower perioperative blood losses after LMW-HES 130/0.4 versus MMW-HES 200/0.5 (1301 ± 551 versus 1821 ± 1222 ml) (*P* < 0.05) in cardiac surgery. Kasper *et al*.[13] examined the influence of unequal doses of LMW-HES 130/0.4 (50 ml/kg) and MMW-HES 200/0.5 (33 ml/kg + add on gelatin) on chest tube drainage. They found no differences despite the substantially higher LMW-HES 130/0.4 dose, signifying that a 50% dose increase compared with MMW-HES 200/0.5 did not deteriorate coagulation and blood loss. Boldt *et al*,[14] showed higher blood losses and more impaired activated thrombelastography measurements after hetastarch in a balanced solution compared with LMW-HES 130/0.4.

Non-hemic colloids, which are used for volume replacement, not only affect the systemic haemodynamics but also influence the microcirculation, pulmonary function, rheology and coagulation. The HES decrease the coagulation factor levels and platelet function beyond that observed by haemodilution alone.[2] It is hypothesized that HES precipitate certain coagulation factors (factor VIII and fibrinogen) making them unavailable to coagulation cascade. Gelatins impair haemostasis by interfering with vWF.[15] In a study it was found that after infusion of gelatin, vWF levels decrease by 32%.[16] Our study shows that after infusion of gelatin there was a significant decrease in vWF levels, but after 6 hours postoperatively, vWF levels increase above the baseline values. HES solutions interfere with blood coagulation with respect to molecular weight, degree of substitution and C2/C6 ratio by an interaction with factor VIII, vWF and platelet dysfunction.[17,18] Our study demonstrated that MMW-HES 200/0.5 causes significant (*P* < 0.05) lowering of vWF as compared to LMW-HES 130/0.4. The recovery of this factor to baseline levels does not occur to the same degree with MMW-HES as compared to LMW-HES 130/0.4 and gelatin. Its levels increase above the baseline values with LMW-HES 130/0.4 and gelatin 6 hours postoperatively. This probably indicates vascular endothelial recovery. HES impair haemostasis by various mechanisms. By their coating effect, large molecules interfere with the function of vWF and hence factor VIII. They also interfere with fibrin formation and platelet function.[19‐23] Thus, the thrombus formed is less stable and more susceptible to lysis.[24‐27] It is speculated that molecular weight of HES is an important factor in determining their effect on blood coagulation. Some investigations have suggested that LMW-HES 130/0.4 may have less effect on coagulation than MMW-HES 200/0.5. Gallandt-Huet[12] *et al*. compared LMW-HES (130/0.4) with MMW-HES (200/0.5) in patients scheduled for CABG. Both HES solutions were used for acute normovolemic haemodilution, for priming the extracorporeal circuit, and for intra and postoperative volume substitution. vWF increased more in the LMW-HES 130/0.4 treated patients than in the patients in whom standard MMW-HES 200/0.5 was given. Blood loss, as well as the use of packed red blood cells, was lower in the LMW-HES 130/0.4 patients indicating considerable benefits with LMW-HES 130/0.4. Similar findings are obtained in our study that patients treated with LMW-HES 130/0.4 have significantly less bleeding as compared to those treated with MMW-HES 200/0.5. Gelatins impair haemostasis also by inhibiting platelet aggregation.[28,29] Haisch *et al*.[30] compared volume replacement with gelatin and LMW-HES 130/0.4; there were no significant differences between the two groups indicating that this LMW-HES 130/0.4 can be safely used in cardiac surgery. All these studies[31‐33] were done in patients scheduled for CABG using the conventional CPB where several other mechanisms related to CPB can be responsible for derangement of coagulation. In our study the deleterious effects of CPB are avoided and it was found that HES with higher molecular weight and degree of substitution cause more impairment of coagulation system. All colloid infusions cause a fall in plasma levels of clotting factors because of haemodilution and impair haemostasis. In our study we have found that PT, PTT are prolonged after colloid infusion which can be explained by haemodilution. Although postoperative bleeding was almost similar in groups II (LMW-HES 130/0.4) and III (GEL), a significantly higher volume of succinylated gelatin was needed to be infused for intravascular volume maintenance, which indicates a weaker intravascular volume expansion effect of gelatins. In group I (MMW-HES 200/0.5) significantly higher volume was infused as compared to group II (LMW-HES 130/0.4) probably because of higher blood loss in group I (MMW-HES 200/0.5).

In a prospective study which compared 3.5% urea-linked gelatin and MMW:HES 200/0.5 in cardiac surgical patients, the total blood loss was higher in HES group resulting in increased use of allergenic blood.[34] Our study demonstrates that MMW-HES 200/0.5 causes significant (*P* < 0.05) lowering of vWF as compared to LMW-HES 130/0.4 and succinylated gelatin. The recovery of this factor to baseline levels dose not occur to the same degree with group I (MMW-HES 200/0.5) as compared to group II (LMW-HES 130/0.4) and gelatin group III (GEL); its levels increase beyond the baseline levels with LMW-HES 130/0.4 and gelatin 6 hours postoperatively. A prospective study with LMW-HES 130/0.4 and gelatin in cardiac surgical patients undergoing coronary revisualization did not demonstrate a difference in blood loss in the two groups; large dose of aprotinin was used in both the groups and use of aprotinin might have blunted the negative effects of colloids on blood loss.[35] LMW-HES 130/0.4, in moderate doses, did not show impaired haemostasis in patients undergoing major abdominal surgery and appears to be safe alternative plasma substitute for intravascular volume replacement. Our data suggest that LMW-HES 130/0.4 is a better alternative to both MMW-HES 200/0.5 and gelatin in patients undergoing OP-CABG because it causes least impairment of coagulation and is a good colloid for intravascular volume maintenance. In addition, there is evidence to show that LMW-HES 130/0.4 preserved endothelial haemostatic repair to after cardiac surgery. Intravascular volume replacement with LMW-HES 130/0.4 reduces inflammatory response due to an improvement in microcirculation with reduced endothelial activation and less endothelial damage.[36]

### Limitation of the study

Though significant findings were found in terms of influence of colloid infusion on coagulation in patients undergoing OP-CABG, the authors recognize that small patient population in the study is a limitation of the study.

## CONCLUSION

6% LMW-HES-130/0/4 has less influence on coagulation parameters as compared to MMW-HES 200/0.5 and modified fluid gelatin solution, when infused in OP-CABG patients.
